# Low‐pH seawater alters indirect interactions in rocky‐shore tidepools

**DOI:** 10.1002/ece3.8607

**Published:** 2022-02-12

**Authors:** Brittany M. Jellison, Kristen E. Elsmore, Jeffrey T. Miller, Gabriel Ng, Aaron T. Ninokawa, Tessa M. Hill, Brian Gaylord

**Affiliations:** ^1^ 3067 Department of Biological Sciences University of New Hampshire Durham New Hampshire USA; ^2^ 8789 Bodega Marine Laboratory University of California Davis Bodega Bay California USA; ^3^ Minnesota Supercomputing Institute University of Minnesota Minneapolis Minnesota USA; ^4^ 160868 Smithsonian Environmental Research Center Edgewater Maryland USA; ^5^ Marine Invasions Laboratory Estuary Ocean Science Center Tiburon California USA; ^6^ 8789 Department of Earth and Planetary Sciences University of California Davis Davis California USA; ^7^ 8789 Department of Evolution and Ecology University of California Davis Davis California USA

**Keywords:** behavior, field study, ocean acidification, predator‐prey, tidepool, trait mediated indirect interactions

## Abstract

Ocean acidification is expected to degrade marine ecosystems, yet most studies focus on organismal‐level impacts rather than ecological perturbations. Field studies are especially sparse, particularly ones examining shifts in direct and indirect consumer interactions. Here we address such connections within tidepool communities of rocky shores, focusing on a three‐level food web involving the keystone sea star predator, *Pisaster ochraceus*, a common herbivorous snail, *Tegula funebralis*, and a macroalgal basal resource, *Macrocystis pyrifera*. We demonstrate that during nighttime low tides, experimentally manipulated declines in seawater pH suppress the anti‐predator behavior of snails, bolstering their grazing, and diminishing the top‐down influence of predators on basal resources. This attenuation of top‐down control is absent in pools maintained experimentally at higher pH. These findings suggest that as ocean acidification proceeds, shifts of behaviorally mediated links in food webs could change how cascading effects of predators manifest within marine communities.

## Graphical abstract

Ocean acidification is expected to degrade marine ecosystems, yet most studies focus on organismal‐level impacts rather than ecological perturbations. Here we address such connections within a rocky shore community, focusing on a three‐level food web involving the keystone sea star predator, *Pisaster ochraceus*, a common herbivorous snail, *Tegula funebralis*, and a macroalgal basal resource, *Macrocystis pyrifera*. We demonstrate that during nighttime low tides, experimentally manipulated declines in seawater pH suppress the anti‐predator behavior of snails, bolstering their grazing, and diminishing the top‐down influence of predators on basal resources.

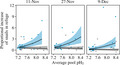

## 1 INTRODUCTION

The ocean is changing at an accelerating rate due to anthropogenic impacts, and many of these changes will detrimentally affect ecosystems (Harley et al., 2006; Poloczanska et al., 2013). Laboratory studies demonstrate that components of ocean change can have adverse effects at the organismal level (Byrne, 2011; Hofmann & Todgham, 2010; Kroeker et al., 2010; Parmesan, 2006; Ries et al., 2009), and these modifications can propagate through systems via species interactions (Connell et al., 2017; Gaylord et al., 2015; Hall‐Spencer et al., 2008; McCoy et al., 2016; Nagelkerken & Munday, 2016). Yet, attempts to identify vulnerable higher‐level ecological processes that depend on such interactions and have potential leverage on marine communities remain underrepresented in experimental work, especially in field efforts (Gaylord et al., 2015; Goldenberg et al., 2017).

Human‐induced global changes to seawater chemistry – often termed “ocean acidification” (a term encapsulating multiple shifts to the marine carbonate system, including shifts in pH as well as alterations to aqueous CO_2_ levels and bicarbonate and carbonate concentrations; Caldeira & Wickett, 2003) – can affect the ecology of marine organisms. Such ecological effects arise both through detrimental impacts on physiology, morphology, and behavior of individual organisms, as well as through more complicated mechanisms tied to altered relationships among species (Clements & Hunt, 2015; Kroeker et al., 2010, 2014; Lord et al., 2017; Pörtner, 2008). For instance, ocean acidification influences the ability of marine organisms to regulate their internal chemistry, calcify, grow, and survive (Portner et al., 2004). Reduced‐pH seawater also alters the behaviors of a variety of taxa, including marine invertebrate species (Clements & Comeau, 2019; Clements & Hunt, 2015; Draper & Weissburg, 2019; Jellison & Gaylord, 2019; Jellison et al., 2016). Because behavior often underlies the functional role of organisms in communities and mediates important species interactions (Clements & Hunt, 2015; Wong & Candolin, 2015), this latter class of effect has crucial relevance to understanding higher‐order ecological consequences of ocean acidification.

Food webs, and the foraging activities and predator‐prey interactions that underlie them, provide a clear avenue by which behavioral changes can alter trophic relationships and community structure (Goldenberg et al., 2017; Ripple & Beschta, 2004; Wootton, 2002). Attention in ecology has targeted, in particular, the potential for predators to influence not only their immediate prey, but also to impose “indirect effects” that cascade vertically across one or more additional trophic levels below, such that a predator affects both its own prey as well as the basal resource that serves as food for the prey. These indirect effects arise through two main routes (Werner & Peacor, 2003). The first, and simpler, route occurs when predators consume prey, which decreases the number of prey feeding on lower trophic levels. Such “density‐mediated indirect interactions” (DMIIs) therefore represent a case where predators affect a basal resource by altering the abundance (or density) of an intermediate consumer. The second pathway arises when a predator alters the behavioral, physiological, or morphological traits of prey (often by inducing fear; Ng & Gaylord, 2020) and consequently reduces rates of foraging of the intermediate consumer on the basal resource (“trait‐mediated indirect interactions” [TMIIs]).

Although both pathways of indirect effects (i.e., DMIIs and TMIIs) have important consequences for community dynamics (Peacor & Werner, 2001; Wootton, 1993), the extent to which ocean acidification might change such interactions remains poorly understood (Allan et al., 2013; Gaylord et al., 2015; Jellison et al., 2016; but for ocean acidification and TMIIs see Lord et al., 2017; Jellison & Gaylord, 2019; and for ocean acidification and non‐trophic indirect effects see Alsterberg et al., 2013; Poore et al., 2013; Garrard et al., 2014). This limitation derives primarily from a shortage of studies that include more than one trophic level and the fact that relatively few experiments manipulate variables in the field where nuances of natural conditions are considered (Jellison & Gaylord, 2019; Riebesell & Gattuso, 2015; Sorte & Bracken, 2015).

Intertidal rocky‐shore environments provide a fitting system to examine indirect effects and other complexities in the field, given that many factors that can be difficult to manipulate in the laboratory vary naturally, including temperature, dissolved oxygen, emergence time, and the abundance or diversity of interacting taxa (Huggett & Griffiths, 1986; Jellison et al., 2016; Morris & Taylor, 1983; Silbiger & Sorte, 2018). Indeed, both the environmental variation and biomass within these areas can be substantially greater than in open‐ocean locations. On the west coast of North America, for instance, nearshore environments are characterized by seasonal upwelling that causes intermittent exposure of intertidal organisms to elevated CO_2_ waters characterized by much lower pH (Chan et al., 2017; Feely et al., 2008; Kroeker et al., 2016). For tidepool organisms, these conditions can be further exacerbated during low tides that occur at night when respiratory carbon released by resident organisms accumulates in pools, as they are isolated from the adjacent ocean. Under such conditions, pH can drop to 7.2 or below even in large tidepools (Huggett & Griffiths, 1986; Jellison et al., 2016; Kwiatkowski et al., 2016; Silbiger & Sorte, 2018). In contrast, pH levels in the open ocean are not expected to decrease to comparable levels even by the year 2100 or beyond (Caldeira & Wickett, 2003; Hauri et al., 2009, 2013).

Here we use a rocky‐shore tidepool system to explore community‐level impacts of low‐pH seawater that occurs naturally in these habitats, and which may also be exacerbated under ocean acidification. We conducted a manipulative field study to investigate how altered seawater chemistry modifies species linkages within a three‐level food web, asking the following three questions: (1) Does reduced pH in natural tidepools impair anti‐predator behavior of prey? (2) Does such impaired behavior lead to increased consumption of prey by predators? (3) Does low pH alter the indirect effects of predators? That is, does the presence of predators alter rates at which prey consume their food, and do such changes differ depending on tidepool pH? To investigate these questions, we used replicate tidepools that include natural habitat and species assemblages, across which we randomly manipulated the presence of predators and carbonate chemistry parameters. Our findings demonstrate that low pH can influence the importance of top‐down predator effects in the field.

## 2 METHODS

### 2.1 Study site and tidepool characteristics

Our study was conducted in rock pools located in Horseshoe Cove in Bodega Bay, California, within the Bodega Marine Reserve of the University of California, Davis. The tidepools are located in the mid to high intertidal zone (shore level range of 0.73–1.46 m above mean lower low water [MLLW]) and have volumes ranging from 1.5 to 14.5 L (substratum surface areas from 0.07 to 0.35 m^2^). Field surveys of the tidepools demonstrate that the dominant space occupiers in the majority of pools are fleshy algae, mussels, and bare rock/rubble (Figure S1). The nearshore seawater that immerses these pools during high tide during the fall season is characterized by temperatures ranging from 9 to 13°C, oxygen values from 6 to 10 mg/L, salinity near 33.5 ppt, and pH values between 7.8 and 8.2 (Figures S4–S6; see also Silbiger & Sorte, 2018). Values of oxygen and pH within the rock pools then fluctuate away from nearshore values during periods of isolation from the adjacent ocean, reaching as low as 0.6 mg/L and 7.0 pH units during nighttime low tides when respiration of resident organisms dominates pool biogeochemistry (Jellison & Gaylord, 2019; Jellison et al., 2016; Kwiatkowski et al., 2016; Silbiger & Sorte, 2018).

### 2.2 Food web

Within the rock pools, we focused on interactions occurring over hours between the keystone sea star predator, *Pisaster ochraceus* (the ochre sea star), the abundant herbivorous black turban snail, *Tegula funebralis*, and the giant kelp, *Macrocystis pyrifera*. *Pisaster ochraceus* ranges from Alaska to Baja California, Mexico, and is the major predator of black turban snails in areas of overlap (Paine, 1969). *Tegula funebralis* grazes on micro‐ and macroalgae within tidepools and is a prominent resident on rocky shores from Vancouver Island, Canada, to Baja California, Mexico. *Macrocystis pyrifera* is a perennial kelp that occurs in large stands and is distributed in the northeast Pacific Ocean along the coasts of North and South America, in South Africa, Australia, New Zealand, and around the sub‐Antarctic islands. Although this species rarely grows in tidepools, it is a preferred food item for *T*. *funebralis* and is consumed after it becomes detached from its growing location and enters intertidal habitat as drift algae (Steinberg, 2013). Therefore, in our system, this algal basal resource operates as a constituent of the detrital compartment of the food web.

In rock pools under ambient seawater conditions, *T*. *funebralis* demonstrates a strong flight response when confronted with waterborne chemical cues from *P*. *ochraceus* (Bullock, 1953; Feder, 1963; Jellison et al., 2016). Upon detection of such cues, *T*. *funebralis* individuals crawl to and up the sides of tidepools until they exit the water where they experience refuge from predation by sea stars (Jellison et al., 2016). This anti‐predator response in snails has been shown to influence densities of algae over short and long timescales, because such behavioral changes result in snails spending more time outside of pools where they do not tend to forage, and do not have access to the resident or drift algae within those pools (Gravem & Morgan, 2016, 2019; Morgan et al., 2016). Thus, the intensity of anti‐predator behavior in snails can modify indirect effects of *P*. *ochraceus* on basal resources in the pools, including both primary producers and detrital constituents.

### 2.3 Experiment overview

To investigate how carbonate system alterations that align with ocean acidification (which for brevity we henceforth refer to as shifts in pH) influence trophic links among sea stars, snails, and algae in the field, we manipulated seawater pH and predator presence in 30 mid to high intertidal rock pools. Following this initial alteration of pH, we measured environmental conditions, prey behavior, the outcome of predator‐prey interactions, and indirect effects on algal consumption at 2‐h intervals through the natural tidal cycle (over a 6–8 h period). We conducted this experiment over nighttime trials on November 11, 2017, November 27, 2017, and December 9, 2017, when tidepools were naturally acidified by respiration of resident organisms. For each trial, treatments were randomized across the 30 tidepools (*N* = 5 for each of three pH treatments crossed with two predator conditions). Our focus was on short‐term responses, and we emphasize that understanding longer term consequences will require further study (Bracken et al., 2018).

### 2.4 Species collections

Ochre sea stars were collected for subsequent deployment in field trials from mid‐intertidal pools in Horseshoe Cove in Bodega Bay, California, two weeks prior to the first field trial. They were then held in running seawater in the laboratory until used in the experiments. For each of the three trials, sea stars were re‐collected from their intertidal pools at the end of the low tide and brought back into the laboratory, placed in flow‐through water, and fed black turban snails *ad libitum* until the next trial. *Macrocystis pyrifera* fronds were collected from Bodega Harbor, California, one day prior to each trial. *Tegula* used in the experiments were those living naturally in the replicate pools.

### 2.5 Carbonate chemistry treatments

**FIGURE 1:**
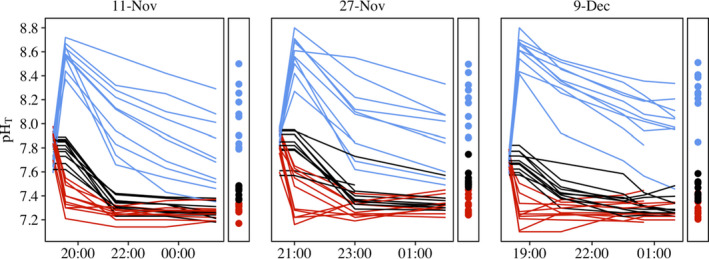
Trajectories through time of pH (total scale) in replicate experimental tidepools for each of three trials in November and December 2017. Lines represent time series of pH for individual pools across the three pH treatments. Raised pH (blue), natural pH (black), and reduced pH (red). Points represent the average pH over the nighttime tidal period for each pool after chemical additions

To investigate how seawater pH and associated carbonate chemistry influences food web dynamics, we employed three pH treatments (raised, reduced, and natural) in field tidepools over three separate nighttime low tides. At the beginning of the low tide, each treatment level was imposed on multiple replicate rock pools (*N* = 10 for each treatment level), after which pool chemistry was allowed to follow its natural progression (Figure 1). We initiated pH manipulation of each pool at the onset of its isolation from adjacent coastal waters; however, starting values of pH before manipulation ranged from 7.6 to 8.0 across all pools. An elevation of ~0.7 pH units was used for the raised‐pH treatment, resulting in starting pH values of 8.3 to 8.8. This treatment was accomplished by raising the starting alkalinity of the pools via chemical additions of NaOH (addition of ~500 μmol alkalinity; Figure S2; following similar methods used on coral reefs; Albright et al., 2016). This treatment was chosen to simulate conditions that can occur in natural producer‐dominated intertidal pools during daytime low tides when photosynthetic activity of algae results in the uptake of dissolved inorganic carbon and therefore increases in pH. On this latter point, it is important to note that NaOH‐induced increases in pH are not accompanied by declines in dissolved inorganic carbon, such that this manipulation does not perfectly mirror photosynthesis‐driven changes. Although work to date suggests pH decreases per se are the driver of behavioral impairments under ocean acidification, further study is warranted (Clements & Hunt, 2015). For reduced‐pH pools, we modified the initial chemistry to be ~0.5 below natural levels to mimic conditions expected for coming decades during nighttime low tides. This pH treatment was established using equimolar additions of HCl and NaHCO_3_, which induces changes in seawater chemistry that duplicate those arising from bubbling CO_2_ directly into the pools (Riebesell et al., 2010; Schulz et al., 2009). This treatment resulted in starting pH values for reduced‐pH pools ranging from 7.1 to 7.6. In the remaining ten pools the initial chemistry of the seawater was not modified (natural‐pH treatment; starting pH ranging from 7.6 to 8.0). A YSI ProPlus sensor was used to measure in situ pH, temperature, salinity, and dissolved oxygen during each sampling period for all pools. These pH measurements were calibrated to the total scale (Riebesell et al., 2010) by means of discrete water bottle samples collected from each pool during the first and last sampling periods (see also Jellison et al., 2016; Jellison & Gaylord, 2019). The bottle samples were analyzed for pH on the total scale via a Shimadzu spectrophotometer and total alkalinity using Gran titration, standardized using certified reference material (A. Dickson Lab, Batch 170; Riebesell et al., 2010). Standard deviation of titrations with reference material was 1.95 μmol/kg sw (*N* = 10) and average standard deviation of all duplicates was 3.60 μmol/kg sw (*N* = 235). In estimating associated carbonate system parameters, we used the seawater chemistry package, Seacarb (v3.3.0; Gattuso et al., 2021) in R 4.1.2 (R Core Team, 2021), with K1 and K2 as quantified by Lueker et al. (2000), *K*_f_ from Dickson and Riley (1979), and K_s_ from Dickson (1990).

### 2.6 Food web treatments

To assess the influence of pH on the strength of snail foraging activity, 10 cm diameter circular pieces of *M*. *pyrifera* blades were secured to polyvinylchloride discs using a hose clamp and placed in the deepest position of each tidepool, for subsequent quantification of the amount of algae grazed. In addition, to determine the influence of tidepool pH on the cascading effects of predators, one sea star (average length center to longest arm = 85 ± 4 mm across 15 individuals) was added at the beginning of the low tide to each of five randomly selected pools from each of the three pH treatments. The other half of the 10 pools from each pH treatment served as no‐predator controls (*N* = 5 for each pH and predator treatment combination). Snail density was not modified in any of the pools. However, to aid in subsequent quantification of snail behavior, immigration and emigration of snails was limited through a movement barrier placed 15 cm above the water line surrounding each pool. The barrier was created using a 5 cm wide epoxy band coated in a nontoxic sticky agent (Tanglefoot) that discourages the transit of crawling gastropods (Aquilino & Stachowicz, 2012; Geller, 1991).

### 2.7 Assessment of trophic links

#### 2.7.1 Behavioral assessment

During the low tide, snail behavior was quantified by recording refuge use by snails of each pool every two hours. Black turban snails normally flee the water within several minutes upon detection of cue from *Pisaster* (Jellison & Gaylord, 2019; Jellison et al., 2016), and we measured predation refuge as the occupancy by snails of the space above the waterline and within the bounds of the epoxy barrier (15 cm above the waterline). This refuge metric was quantified for each pool as the maximum proportional increase in snails out of water in comparison to the first time point, divided by the initial count of snails out of water. The number of snails in refuge for the first time point was recorded prior to the addition of predators or chemical manipulation of tidepool waters and serves as a baseline.

$$\frac{\text{count of snails out of water at time period}\;i‐\text{initial number of snails out of water}}{\text{initial number of snails out of water}}$$

This metric yielded a relative scale with zero being no change in snail behavior within a given tidepool and 1.0 indicating a doubling of snails occupying refuge space. This metric was used for logistical feasibility, as acquiring an accurate count of snails in and out of a given pool during the night was not possible due to the finite duration of the low tide. However, previous field surveys indicated a strong correlation between snails starting in the water and out of the water for a given pool (*N* = 8 pools, *R* ^2^ = 0.97).

#### 2.7.2 Evaluation of sea star predation on snails

The influence of pH on the outcome of predator‐prey interactions was measured as the total number of snails captured by each sea star. At the end of each trial, sea stars were inverted, and their oral surfaces inspected to determine the number of *Tegula* snails that were gripped firmly by sea star tube feet near the mouth at the center of the disk. This metric was used as a proxy for actual consumption, as handling times for sea stars can extend beyond the low‐tide period (Paine, 1969).

#### 2.7.3 Assessment of indirect predator effects

The influence of pH on the strength of trophic indirect effects was assessed by comparing the amount of algae snails grazed across predator treatments and pH levels. Photographs of algal discs were taken before and after the low tide and analyzed for change in surface area (cm^2^) via Image J software. Changes in algal discs were attributed to grazing by black turban snails as they were the only grazer observed on the algae during the experiment. However, other grazers were present in the pools and could have contributed to the loss in kelp surface area.

### 2.8 Statistical analysis

To assess the influence of tidepool pH on the outcome of trophic interactions, we took as an independent variable the average pH over the nighttime tidal period for each pool. We used the maximum proportional increase in snails out of water during the assessment period for a given pool on a given date as our metric of snail anti‐predator behavior. We assessed then how the presence of a predator, tidepool pH, and date influenced snail behavior using a linear mixed‐effects model (log transformed response variable) with tidepool identity included as a random effect to account for repeated trials across dates as well as natural variation in community composition and geometry (e.g., shape, size, and area‐volume ratios) among pools. The interaction between pH and predator presence was assessed to determine if sea star predators initiated a flight response in snails under all pH conditions. The hypothesis that snail capture was influenced by pH was tested using a generalized linear mixed‐effects model (Poisson GLMM, log‐link) with tidepool pH and date included as independent variables and tidepool identity included as a random effect. The possibility that algal consumption was influenced by pH, predator presence, and date was tested using a linear mixed‐effects model (log transformed response variable) with tidepool identity included as a random effect. The interaction term was then used to determine whether the strength of predator indirect effects on algae was different across tidepool pH levels. Models were fit using the lme4 package (v067; Bates et al., 2015) in the statistical software, R 4.1.2 (R Core Team, 2021). The best‐fitting model for each response variable was selected using the Akaike information criterion (AIC). Visual inspections of *Q*–*Q* and residuals versus fitted plots were used to verify assumptions of the general linear mixed effects models and scaled residuals were inspected to verify assumptions for the generalized linear mixed effects model using the DHARMa package (v0.4.4; Hartig, 2021) in R. To diagnose each of our models for disproportionately influential data that could negatively alter inference, we calculated Cook's distance and tested for changing levels of significance using the influence.ME package (Nieuwenhuis et al., 2012) in R. Only in our snails caught model did our results for Cook's Distance and the changing level of significance both identify a particular pool (pool 26) as being overly influential on the regression outcome regarding the effect of pH. In this case we deleted this pool from the data and re‐evaluated the model.

## 3 RESULTS

### 3.1 Behavioral assay

**FIGURE 2:**
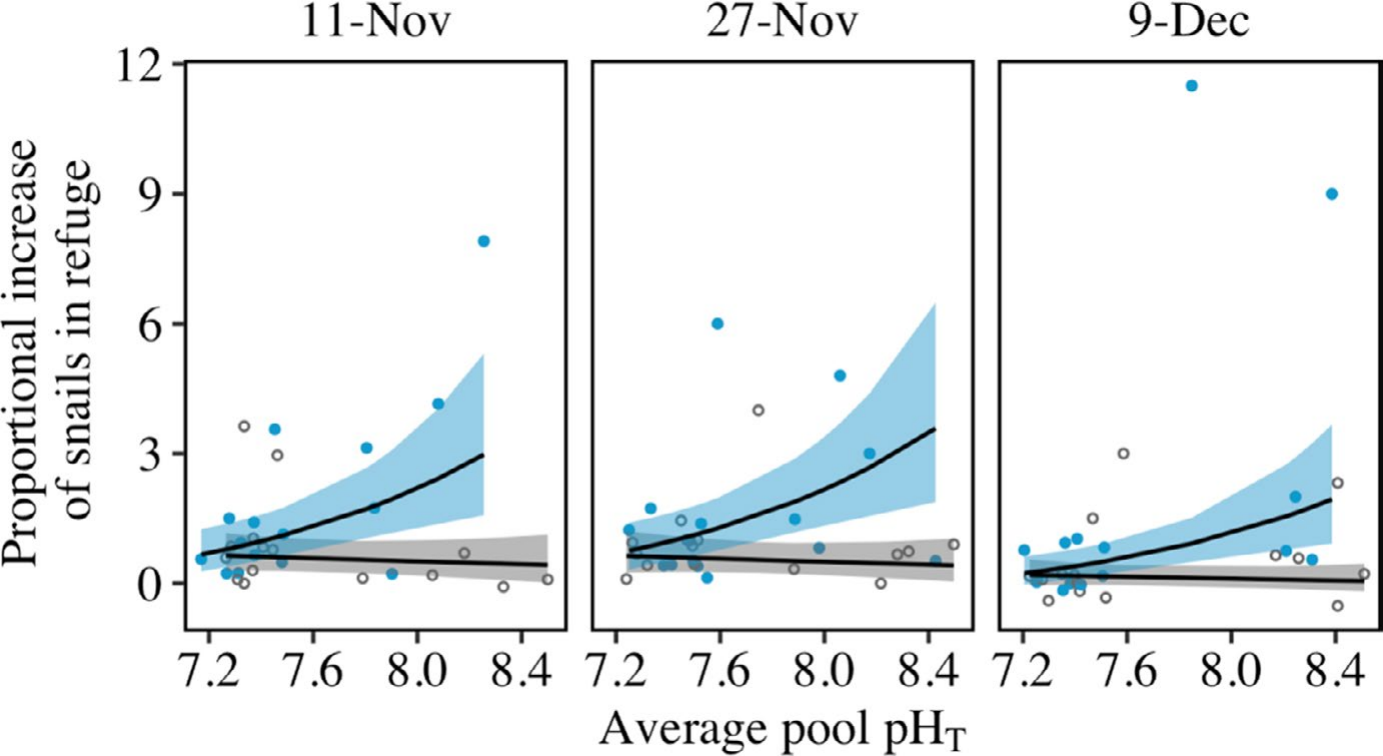
Experimental reduction of tidepool pH impairs the refuge‐seeking behavior of black turban snails (*T*. *funebralis*), causing them to act as if predators are absent. This trend appears as a decline under reduced pH in the maximum proportional increase of snails out of water in the presence of the sea star *Pisaster ochraceus* (convergence of the with‐predator [blue] and without‐predator [gray] trend lines). Panels indicate data from three nighttime trials and pH is calculated as the nightly average pH for each pool. Lines are based on a linear mixed‐effects model of log‐transformed data and shading represents 95% confidence intervals. Note that a *y*‐axis value of 1 represents a doubling of snails using refuge

Reduced pH in rock pools during nighttime low tides attenuated the reaction of snails to predation risk. In high‐pH conditions, snails responded to the presence of a sea‐star predator by fleeing the water. Indeed, predator presence caused increases of up to 350% in refuge use by snails. However, proportional responses of snails to predator cue were attenuated in low‐pH tidepools. For example, we observed a threefold reduction under low pH compared to high pH in proportional refuge responses of snails exposed to predator cue (Figure 2; decline of refuge use metric by 164% as pH dropped one unit; model prediction for effect of pH in the presence of a predator). No analogous reduction in refuge use occurred in the absence of predator cue as refuge use was uniformly low, resulting in a significant interaction between pH and predator treatment (Figure 2, Tables S7 and S10; *p* = .01). These findings match those of prior laboratory mesocosm experiments (Jellison & Gaylord, 2019). Manipulation of starting pH conditions in tidepools did not by itself alter snail behavior, apparent from the absence of any relationship between pH and the fleeing behavior of snails when a predator was absent. Had chemical additions to the tidepools caused physiological impacts or altered behavior of snails, we would have anticipated an effect of pH, even in the absence of predators, on movement of snails into refuge space in pools, which we did not detect.

### 3.2 Evaluation of sea star predation on snails

**FIGURE 3:**
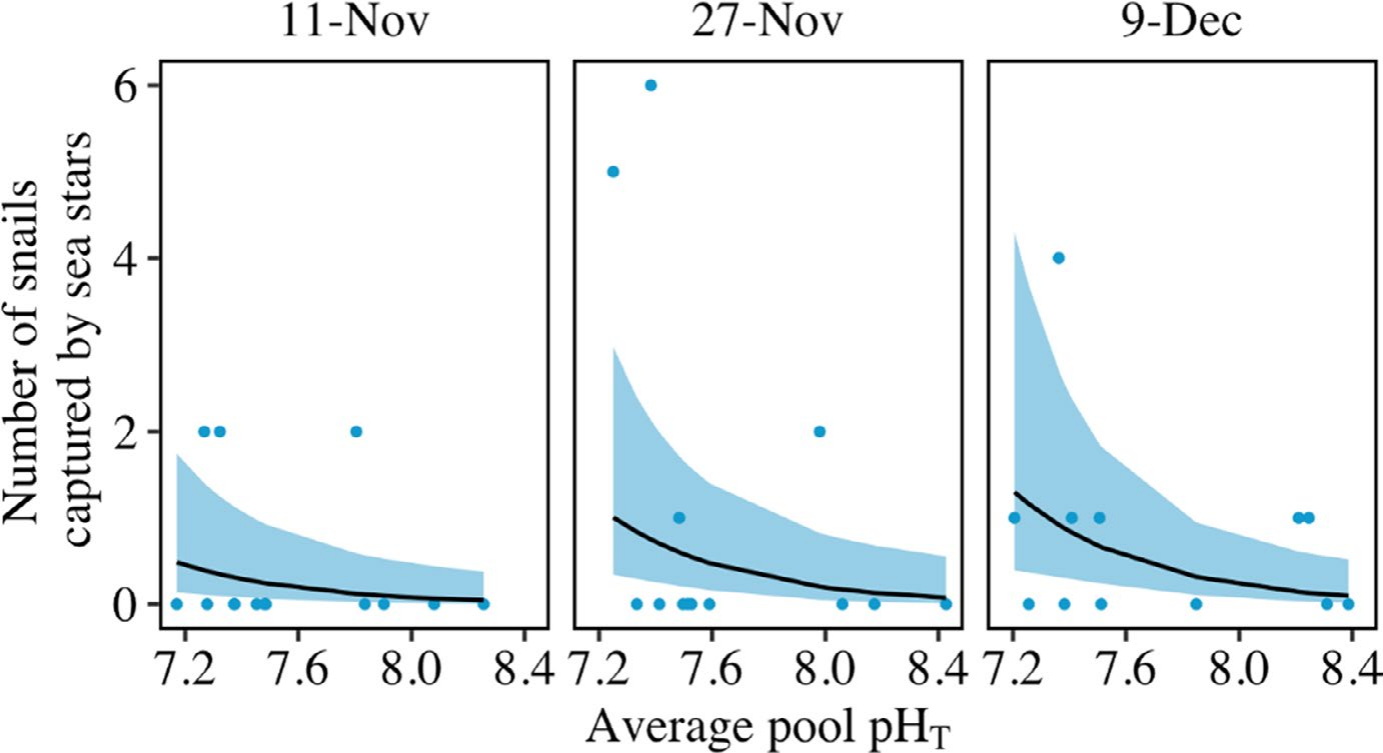
Fewer snails exited the water under low pH which led to increased capture rates by sea stars. Lines represent the number of snails caught by sea stars in pools with predators, based on a generalized linear mixed‐effects model with a log link function (Poisson distribution). Shading represents 95% confidence intervals

The anti‐predator behavior of black turban snails was reduced in low‐pH pools which translated into an increase in predation risk (pH, *p* = .009). The number of snails captured by sea stars was increased in low‐pH pools (Figure 3, Tables S8 and S11; 89% increase in snails captured with one unit decrease in pH). Sea star movement behavior was not evaluated in this study. However, in a previous laboratory study, consumption of snails rose even as the locomotory activity of sea stars decreased under low pH (see Jellison & Gaylord, 2019).

### 3.3 Indirect effects of predators

**FIGURE 4:**
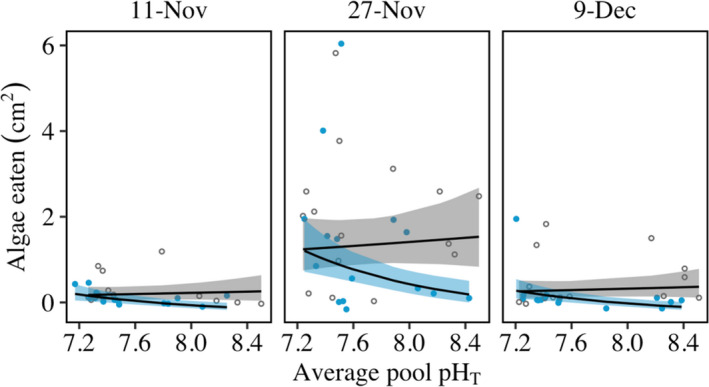
Cascading effects of predators on algal consumption are eliminated under low pH. This outcome is apparent from the convergence of the with‐predator (blue) and without‐predator (gray) trend lines at reduced average pool pH levels. Fitted lines are based on a linear mixed effects model of log‐transformed data, and shading represents 95% confidence intervals

Low‐pH seawater weakened the strength of the behaviorally mediated trophic cascade as seen by a significant interaction between predator presence and pH level (*p* = .003). This effect was driven predominantly by a reduction in the number of snails exiting the water in the low‐pH treatment, which increased the proportion of snails that maintained feeding activities underwater even in the presence of a predator. Thus, while in high‐pH pools snails consumed less algae in the presence of a predator versus without a predator, in low‐pH pools snails consumed a similar amount of algae regardless of the predator treatment (Figure 4; 63% increase in algae eaten by snails in the presence of predators as pH dropped one unit). As a result, the strength of cascading top‐down effects on algae was reduced in low‐pH pools (Figure 4, Tables S9 and S12).

Algal consumption levels also differed among dates, potentially due to natural differences in snail hunger or other time dependence in grazing intensity. Although this trend altered the effect size of pH on algal consumption, reduced pH consistently increased the amount of algae grazed in the presence of a predator regardless of date. In addition, both dissolved oxygen concentration and temperature decreased with time in all pools across the three dates. These factors thus co‐varied with pH in natural, reduced, and raised‐pH pools (all three factors decreased through time). However, because the pattern of covariation differed between treatments (Figures S4 and S5), the manipulation of pH and its consistent relationship to altered behavior and modified indirect interactions allowed these effects to be attributed to shifts in carbonate chemistry as opposed to decreases in temperature or oxygen (also see Jellison & Gaylord, 2019).

## 4 DISCUSSION

During nighttime low tides in natural tidepools, reduced seawater pH impaired anti‐predator responses of black turban snails, thereby diminishing the top‐down influence of predators on lower levels of the food web. Even in the presence of predators, snails in low‐pH pools remained in risky habitat and foraged on algae as if there was not a threat of being eaten. The fact that this study was conducted under field conditions, with real‐world variation in environmental parameters and a full complexity of constituent taxa, highlights the capacity for low‐pH seawater to alter prey behavior and influence trophic links in rocky‐shore marine communities. Although tidepools are not isolated from the adjacent ocean all the time, conditions such as those examined in the study happen routinely during higher‐amplitude tidal cycles, and thus may apply to as many as 40% of dates through a year at our field site. The possibility that low‐pH conditions in tidepools have been influencing *Pisaster*‐*Tegula* dynamics for centuries therefore deserves consideration, in addition to implications of these results for future conditions of ocean acidification where pH levels in tidepools may decline further.

Indeed, the fact that snail anti‐predator behaviors were degraded in both reduced‐pH tidepools and natural‐pH tidepools during nighttime low tides is notable. This outcome suggests that although snails have an evolutionary history of living in tidepools and may be acclimatized to large diel fluctuations in pH, there may also be difficult‐to‐overcome physiological constraints on how low a pH they can tolerate before experiencing behavioral impairment (Briffa et al., 2012; de la Haye et al., 2012). The possibility that a low‐pH limit might exist, beyond which behavioral degradation is unavoidable even in species with longstanding exposure to such extremes, could operate in analogy to previous work focused on climatic warming. This previous work shows that certain other intertidal invertebrates already live near an upper thermal bound that cannot be readily surmounted by acclimatization or adaptation (Somero, 2002; Stillman, 2002). Additional field experiments and comparative studies are also needed to determine if the snail responses observed in our study, which occurred in the face of strongly intermittent low‐pH extremes, also hold if snails experience such fluctuations for periods longer than in our study, or under pH exposures that are temporally less variable than those characteristic of tidepools. Regardless, the present findings suggest a strong potential for seawater chemistry to influence both modern and future species interactions in marine tidepool communities.

We can anticipate that the pH‐induced changes we demonstrate will also be shaped by the consistency with which taxing conditions might be imposed and the presence of other stressors. With routine exposure to acidified conditions and if abundant resident algal resources are present within rock pools (i.e., algae not subject to availability pulses as with *M*. *pyrifera*), elevated CO_2_ might act as a resource for photosynthesizers. In such a situation, bottom‐up effects might have the potential to overshadow the altered outcome of top‐down predator effects we observed in our study (Connell et al., 2017; Goldenberg et al., 2017; Nagelkerken & Connell, 2015; Sorte & Bracken, 2015). Additional manipulative field experiments, including ones spanning weekly to monthly timescales, and that consider other nutrients such as nitrate (Bracken et al., 2018; Sorte & Bracken, 2015) are needed to disentangle the role CO_2_ can play as both a resource and a stressor, and the capacity of ocean acidification to shift the balance between bottom‐up and top‐down forcing in future decades.

Levels of pH can vary strongly in space and time, as is apparent from variation in conditions we observed across our tidepools (Figure 1, natural pools), as well as in other studies (Huggett & Griffiths, 1986; Silbiger & Sorte, 2018). Our results suggest that this natural variability has the potential to drive differences in community composition by influencing the heterogeneity of predator effects within the system. As such, it could contribute to mosaic‐like spatiotemporal variation in function, structure, and diversity across tidepool landscapes within a site or region. Furthermore, as ocean acidification proceeds and the pH of tidepools at the initiation of low tide declines, the heterogeneity in pH conditions could be exacerbated due to varied biophysical feedbacks in response to elevated CO_2_ (Figure 1; Silbiger & Sorte, 2018) which could drive amplified disparity across pools (Briffa et al., 2012).

Ocean acidification effects will be complex, and higher‐level community processes will underlie important consequences for marine ecosystems (Gaylord et al., 2015; Goldenberg et al., 2017). Our results indicate that under field conditions and with all the complexity that those varied conditions imply, top‐down indirect effects of predators can be impaired under reduced seawater pH due to perturbed anti‐predator behaviors in prey. These results reiterate the crucial need for additional field evaluations that isolate the role of ocean chemistry in modifying species interactions within natural marine communities, now and into the future (Kline et al., 2012; Riebesell & Gattuso, 2015).

## AUTHOR CONTRIBUTIONS

**Brittany M. Jellison:** Conceptualization (lead); Data curation (lead); Formal analysis (lead); Funding acquisition (equal); Investigation (lead); Methodology (lead); Project administration (lead); Validation (lead); Visualization (lead); Writing – original draft (lead); Writing – review & editing (lead). **Kristen E. Elsmore:** Data curation (supporting); Investigation (supporting); Methodology (supporting); Writing – review & editing (supporting). **Jeffrey T. Miller:** Data curation (supporting); Investigation (supporting); Methodology (supporting); Writing – review & editing (supporting). **Gabriel Ng:** Data curation (supporting); Formal analysis (supporting); Investigation (supporting); Methodology (supporting); Writing – review & editing (supporting). **Aaron T. Ninokawa:** Data curation (supporting); Investigation (supporting); Methodology (supporting); Writing – review & editing (supporting). **Tessa M. Hill:** Conceptualization (supporting); Methodology (supporting); Resources (supporting); Writing – review & editing (supporting). **Brian Gaylord:** Conceptualization (supporting); Formal analysis (supporting); Funding acquisition (equal); Investigation (supporting); Methodology (supporting); Resources (lead); Supervision (equal); Validation (supporting); Visualization (supporting); Writing – original draft (supporting); Writing – review & editing (supporting).

## Supporting information

Supplementary Material

## Data Availability

The data that support the findings of this study are openly available in Dryad at https://doi.org/10.5061/dryad.sn02v6x5n.
