# Supplementary material for: Low‐pH seawater alters indirect interactions in rocky‐shore tidepools
Source: Ecol Evol. 2022 Feb 12;12(2):e8607. doi: 10.1002/ece3.8607 (PMC8840877; doi:10.1002/ece3.8607)
Supplement: Supplementary file 1 — Supplementary Material [file ECE3-12-e8607-s001.docx]

APPENDICES

APPENDIX A. Methods for tidepool characterization

Thirty small to medium tidepools were chosen within the mid to high intertidal zone of Horseshoe Cove in Bodega Bay, California, within the Bodega Marine Reserve. Each pool was characterized for physical features and biological assemblage. The physical attributes of pools were measured as follows. Tidal height was measured as the predicted tidal height for NOAA site 9415625 at the time of submergence for each pool (range of 0.73 to 1.46 meters above MLLW). Water volume was measured during isolation at low tide using a dye dilution assay (Silbiger 2018, Pfister 1995) where a known volume of McCormick food coloring was added to each pool and mixed well. Resultant dye concentration was determined with a Shimadzu spectrophotometer at 630 nm and used to calculate pool volume (range of 1.5 to 14.5 L). Bottom surface area and water surface area were measured using a flexible mesh net with a 2cm X 2 cm grid (range of 0.07 to 0.35 m^2^ and 0.05 to 0.3 m^2^, respectively). Bottom surface area was defined as all the rock bottom and sides of the pool that were submerged in water during low tide. Maximum depth was quantified using a ruler placed at the deepest location in each pool (range of 7.5 to 27.0 cm).

Community composition of pools was determined through visual surveys during a daytime low tide using a flexible mesh net with a 2cm X 2 cm grid placed along the bottom and sides of each pool submerged in water. Each sessile organism was measured for percent cover and identified down to the lowest possible taxonomic unit. Then each taxon was placed into a group identifier (red algae, microalgae, surfgrass, CCA, mussel bed, anemone, bare rock, and rubble). Mobile invertebrates were counted individually. Two of the pools contained a *Pisaster ochraceus* individual and each was removed prior to the start of the experiment.

*Appendix Figures*

A.


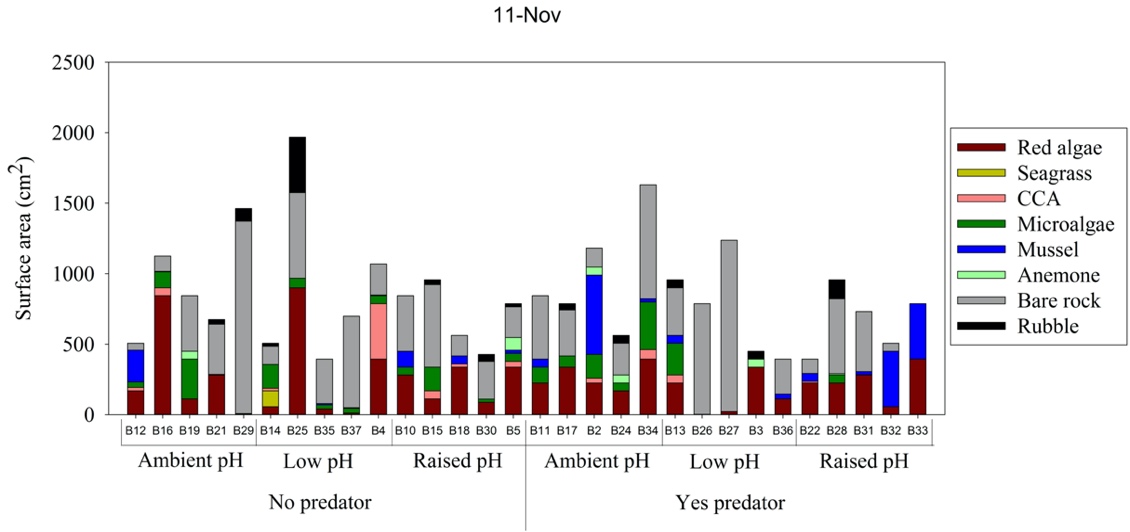


B.


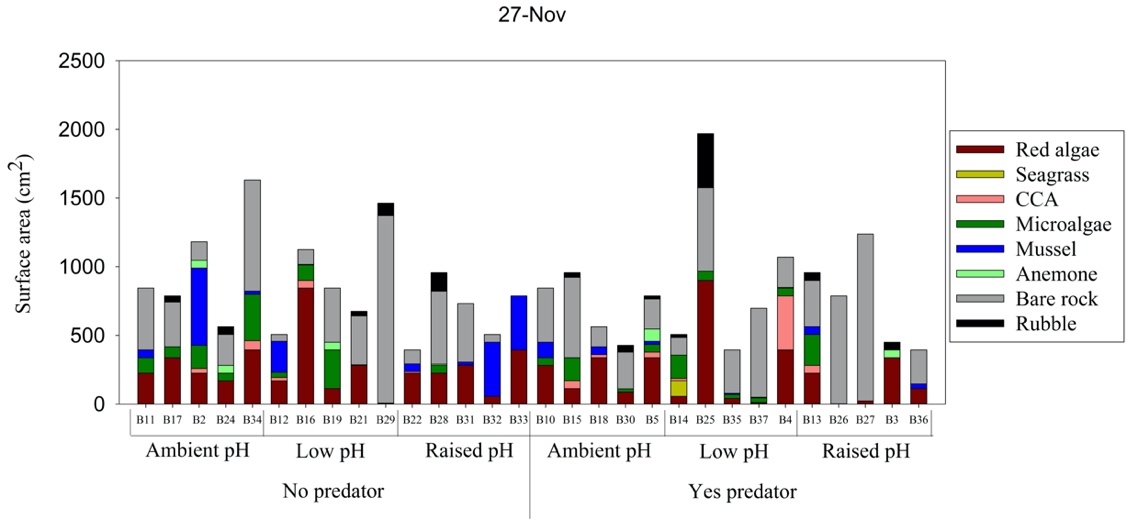


C.


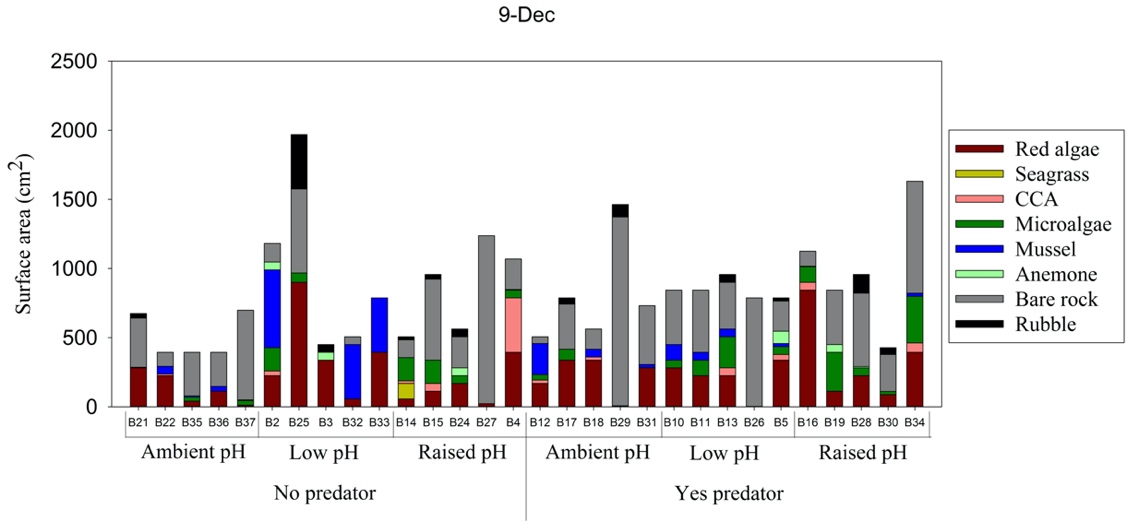


FIGURE S1. Community composition of tidepools. The color of each bar represents the total surface area coverage for each functional group for each tidepool (labeled B1-B36). Treatments were randomized across dates, so each plot depicts the treatment associated with a given pool for each date (A-C). The total bottom surface area for a given pool is depicted by the height of the bar.


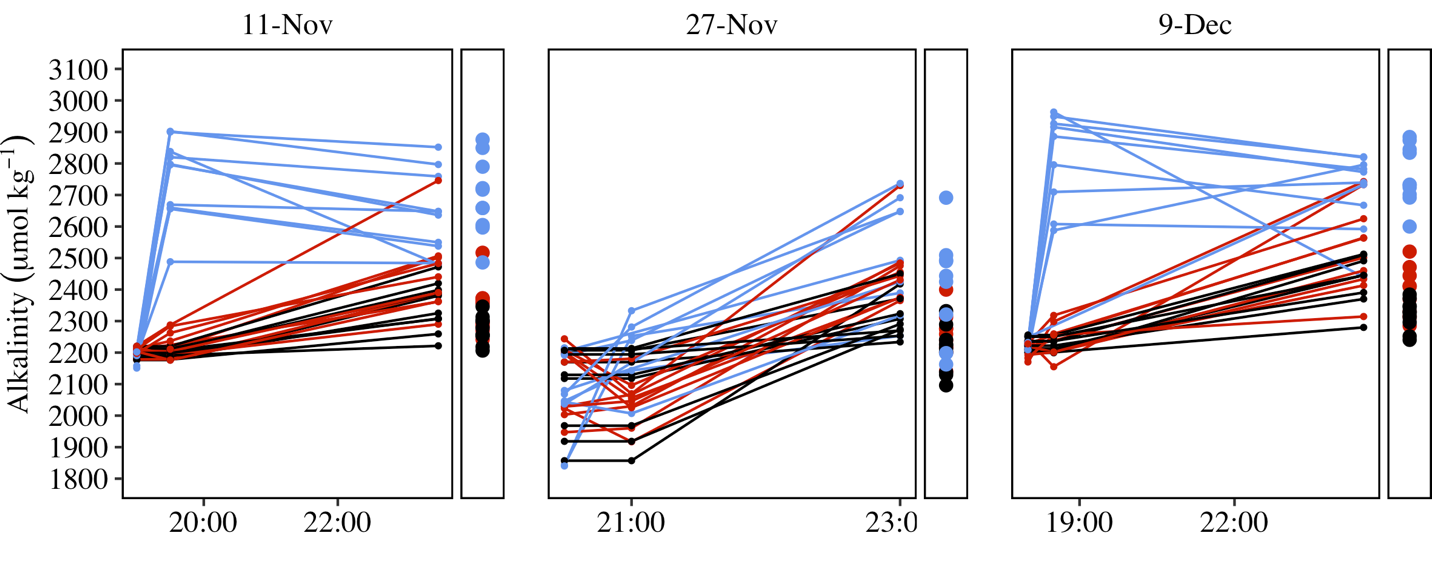


FIGURE S2. A record of alkalinity (μmol/kg sw) in replicate experimental tidepools through time for each trial (panels). Lines represent time series of alkalinity for individual pools across the three pH treatments. Raised pH (blue), natural pH (black), and low pH (red). Points in side panels represent the average alkalinity over the nighttime tidal period for each pool after chemical additions.


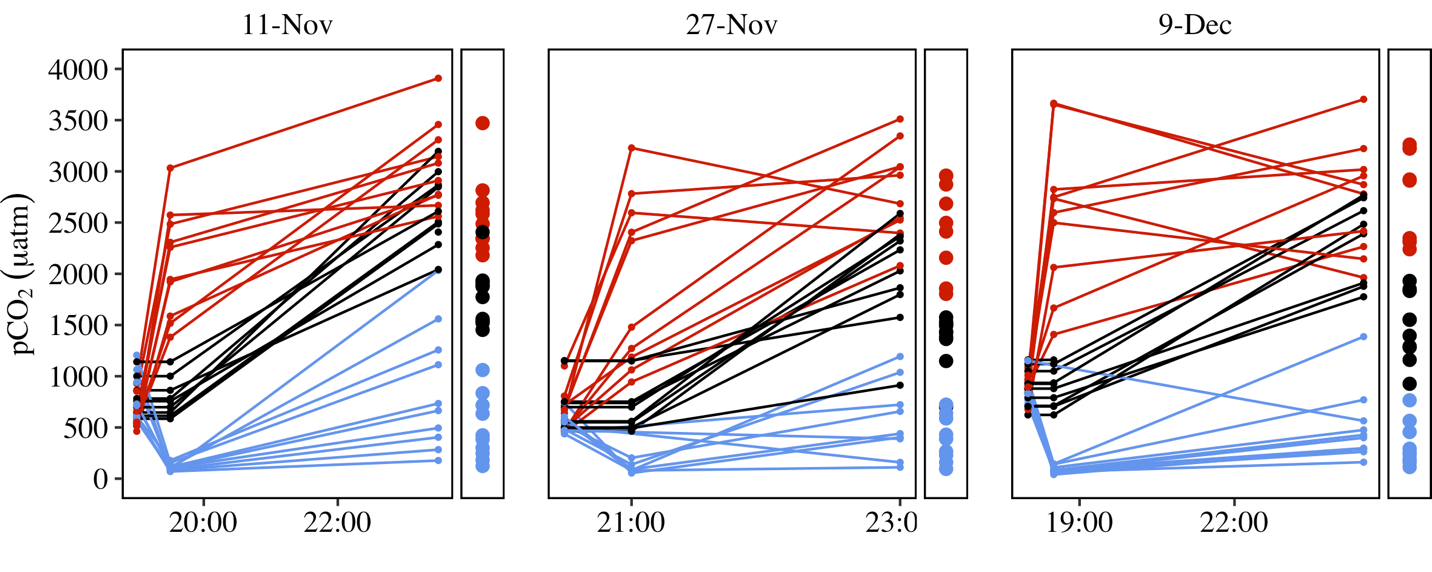


FIGURE S3. A record of pCO_2_ (μatm) in replicate experimental tidepools through time for each trial (panels). Lines represent time series of pCO_2_ for individual pools across the three pH treatments. Raised pH (blue), natural pH (black), and low pH (red). Points in side panels represent the average pCO_2_ over the nighttime tidal period for each pool after chemical additions.


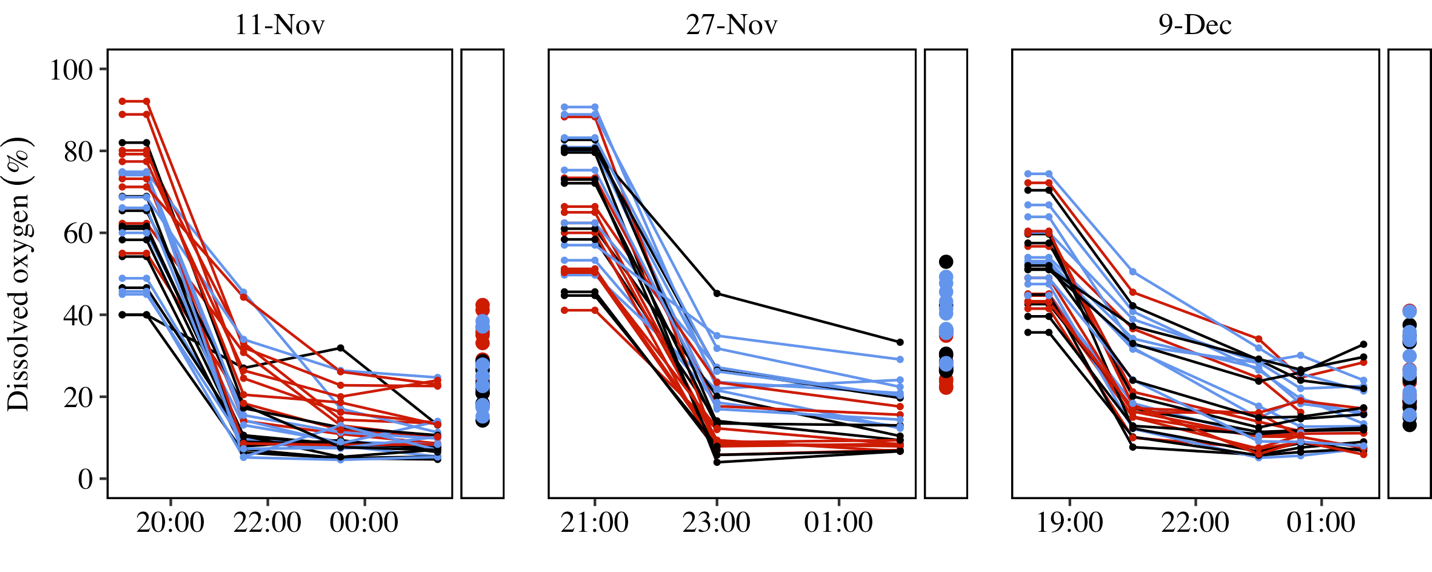


FIGURE S4. A record of dissolved oxygen (% saturation) conditions in replicate experimental tidepools through time for each trial (panels). Lines represent time series of dissolved oxygen for individual pools across the three pH treatments. Raised pH (blue), natural pH (black), and low pH (red). Points in side panels represent the average dissolved oxygen over the nighttime tidal period for each pool after chemical additions.


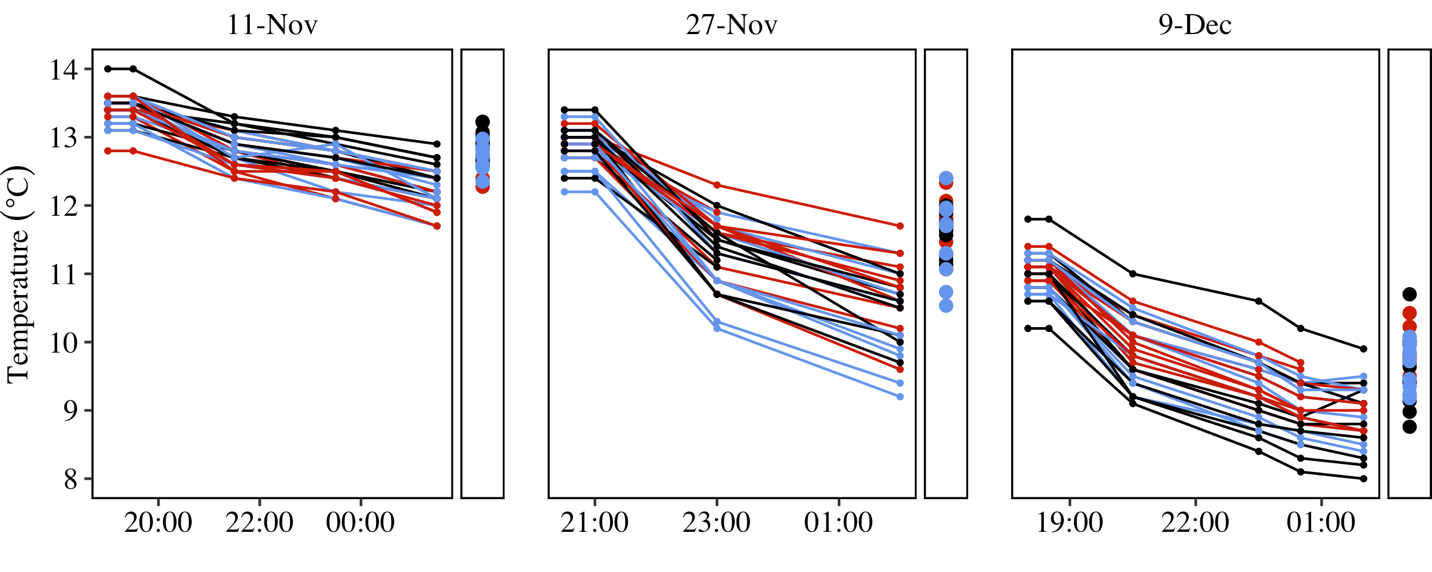


FIGURE S5. A record of temperature (˚c) conditions in replicate experimental tidepools through time for each trial (panels). Lines represent time series of temperature for individual pools across the three pH treatments. Raised pH (blue), natural pH (black), and low pH (red). Points in side panels represent the average temperature over the nighttime tidal period for each pool after chemical additions.


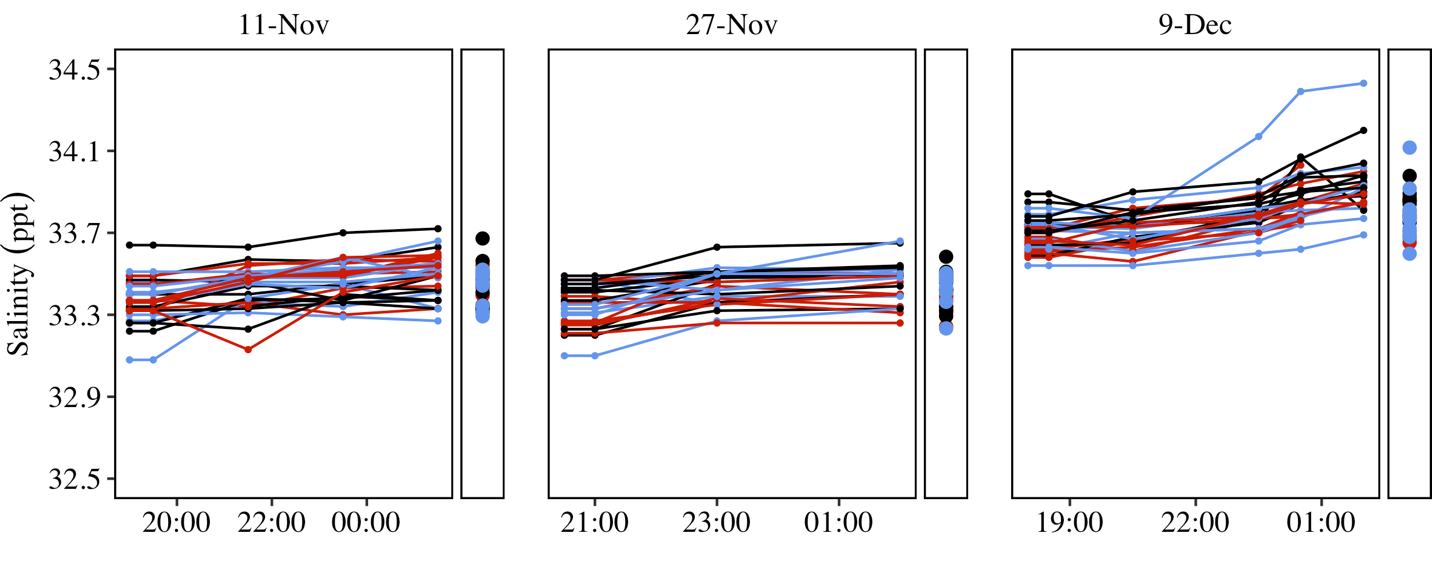


FIGURE S6. A record of salinity (ppt) conditions in replicate experimental tidepools through time for each trial (panels). Lines represent time series of salinity for individual pools across the three pH treatments. Raised pH (blue), natural pH (black), and low pH (red). Points in side panels represent the average salinity over the nighttime tidal period for each pool after chemical additions.

*Appendix Tables*

TABLE S7. Summary results for LMM model testing the effect of predator presence, total pH, and date on the propensity of snails to enter refuge. Fixed effects are shown based on log transformed data. Bold values are statistically significant.

| Parameter | Estimate | Std. error | Est. df | t value | p value |
| --- | --- | --- | --- | --- | --- |
| Intercept (No predator, 11/27/17) | 0.18 | 0.21 | 48 | 0.88 | 0.38 |
| Predator present | 0.003 | 0.25 | 62 | 0.01 | 0.99 |
| pH | -0.16 | 0.26 | 59 | -0.63 | 0.53 |
| Date -11/11/17 | 0.01 | 0.18 | 60 | 0.05 | 0.96 |
| **Date - 12/9/17** | **-0.47** | **0.18** | 42 | **-2.57** | **0.01** |
| **Predator Present*pH** | **1.14** | **0.40** | 64 | **2.87** | **0.01** |

TABLE S8. Summary results for GLMM model testing the effect of pH and date on the number of snails captured by the individual sea star in the predator addition pools. Fixed effects are shown in log space. Bold values are statistically significant.

| Parameter | Estimate | Std. error | t value | p value |
| --- | --- | --- | --- | --- |
| Intercept (11/27/17) | 0.18 | 0.56 | 0.32 | 0.75 |
| **pH** | **-2.20** | **0.84** | **-2.60** | **0.009** |
| Date -11/11/17 | -0.91 | 0.64 | -1.42 | 0.16 |
| Date -12/9/17 | 0.16 | 0.51 | 0.30 | 0.76 |

TABLE S9. Summary results for LMM model testing the effect of predator presence, pH, and date on the amount of algae consumed by snails. Fixed effects are shown based on log transformed data. Bold values are statistically significant.

| Parameter | Estimate | Std. error | Est. df | t value | p value |
| --- | --- | --- | --- | --- | --- |
| Intercept (No predator, 11/27/17) | 0.40 | 0.21 | 83 | 1.94 | 0.06 |
| Predator present | 0.07 | 0.26 | 76 | 0.26 | 0.79 |
| pH | 0.14 | 0.24 | 77 | 0.59 | 0.56 |
| **Date - 11/11/17** | **-1.23** | **0.19** | 55 | **-6.61** | **<0.001** |
| **Date - 12/9/17** | **-1.04** | **0.17** | 54 | **-6.19** | **<0.001** |
| **Predator present*pH** | **-1.14** | **0.37** | 76 | **-3.11** | **0.003** |

TABLE S10. Results of model selection analysis for candidate general linear mixed effects models describing the relationship between proportion increase in snails in refuge, pH, the presence or absence of a predator, and trial date. Parentheses denote random effects. Bold font indicates models with the lowest AIC value.

| Model | Model parameters | AIC |
| --- | --- | --- |
| 1 | Log(Prop. snails in refuge) = 1+(Pool) | 234.11 |
| 2 | Log(Prop. snails in refuge) = pH+(Pool) | 234.8 |
| 3 | Log(Prop. snails in refuge) = Predator presence+(Pool) | 226.7 |
| 4 | Log(Prop. snails in refuge) = Date+(Pool) | 231.1 |
| 5 | Log(Prop. snails in refuge) = pH+Predator presence+(Pool) | 226.9 |
| 6 | Log(Prop. snails in refuge) = pH+Date +(Pool) | 231.4 |
| 7 | Log(Prop. snails in refuge) = Date+Predator presence+(Pool) | 222.5 |
| 8 | Log(Prop. snails in refuge) = pH+Predator presence+Date+(Pool) | 222.2 |
| 9 | Log(Prop. snails in refuge) = pH*Predator presence+(Pool) | 221.1 |
| 10 | Log(Prop. snails in refuge) = pH*Date +(Pool) | 233.9 |
| 11 | Log(Prop. snails in refuge) = Date*Predator presence+(Pool) | 226.0 |
| **12** | **Log(Prop. snails in refuge) = pH*Predator presence+Date+(Pool)** | **216.1** |
| 13 | Log(Prop. snails in refuge) = Predator presence*Date+pH+(Pool) | 225.6 |
| 14 | Log(Prop. snails in refuge) = Date*pH+Predator presence+(Pool) | 224.6 |
| 15 | Log(Prop. snails in refuge) = pH*Predator presence*Date+(Pool) | 224.3 |

TABLE S11. Results of model selection analysis for candidate generalized linear mixed effects models (Poisson GLMM, log-link) describing the relationship between snails captured, pH, and trial date. Parentheses denote random effects. Bold font indicates models with the lowest AIC value.

| Model | Model parameters | AIC |
| --- | --- | --- |
| 1 | Snails caught = 1+(Pool) | 101.8 |
| **2** | **Snails caught = pH+(Pool)** | **96.7** |
| 3 | Snails caught = Date+(Pool) | 104.9 |
| **4** | **Snails caught = pH+Date +(Pool)** | **97.9** |
| 5 | Snails caught = pH*Date +(Pool) | 100.2 |

TABLE S12. Results of model selection analysis for candidate general linear mixed effects models describing the relationship between the amount of algae consumed by snails, pH, the presence or absence of a predator, and trial date. Parentheses denote random effects. Bold font indicates models with the lowest AIC value.

| Model | Model parameters | AIC |
| --- | --- | --- |
| 1 | Log(Algae eaten) = 1+(Pool) | 251.3 |
| 2 | Log(Algae eaten) = pH+(Pool) | 253.1 |
| 3 | Log(Algae eaten) = Predator presence+(Pool) | 248.7 |
| 4 | Log(Algae eaten) = Date+(Pool) | 221.2 |
| 5 | Log(Algae eaten) = pH+Predator presence+(Pool) | 250.3 |
| 6 | Log(Algae eaten) = pH+Date +(Pool) | 221.2 |
| 7 | Log(Algae eaten) = Date+Predator presence+(Pool) | 216.1 |
| 8 | Log(Algae eaten) = pH+Predator presence+Date+(Pool) | 215.7 |
| 9 | Log(Algae eaten) = pH*Predator presence+(Pool) | 250.3 |
| 10 | Log(Algae eaten) = pH*Date +(Pool) | 224.8 |
| 11 | Log(Algae eaten) = Date*Predator presence+(Pool) | 219.3 |
| **12** | **Log(Algae eaten) = pH*Predator presence+Date+(Pool)** | **210.0** |
| 13 | Log(Algae eaten) = Predator presence*Date+pH+(Pool) | 219.0 |
| 14 | Log(Algae eaten) = Date*pH+Predator presence+(Pool) | 219.0 |
| 15 | Log(Algae eaten) = pH*Predator presence*Date+(Pool) | 217.3 |
